# Evolution of tsunami warning systems and products

**DOI:** 10.1098/rsta.2014.0371

**Published:** 2015-10-28

**Authors:** Eddie Bernard, Vasily Titov

**Affiliations:** Pacific Marine Environmental Laboratory, NOAA, Seattle, WA 98115, USA

**Keywords:** tsunami, tsunami warnings, deep-ocean assessment and reporting of tsunamis, tsunami flooding forecasts, tsunami-induced current forecasts, tsunami magnitude scale

## Abstract

Each year, about 60 000 people and $4 billion (US$) in assets are exposed to the global tsunami hazard. Accurate and reliable tsunami warning systems have been shown to provide a significant defence for this flooding hazard. However, the evolution of warning systems has been influenced by two processes: deadly tsunamis and available technology. In this paper, we explore the evolution of science and technology used in tsunami warning systems, the evolution of their products using warning technologies, and offer suggestions for a new generation of warning products, aimed at the flooding nature of the hazard, to reduce future tsunami impacts on society. We conclude that coastal communities would be well served by receiving three *standardized, accurate*, real-time tsunami warning products, namely (i) tsunami energy estimate, (ii) flooding maps and (iii) tsunami-induced harbour current maps to minimize the impact of tsunamis. Such information would arm communities with vital flooding guidance for evacuations and port operations. The advantage of global standardized flooding products delivered in a common format is efficiency and accuracy, which leads to effectiveness in promoting tsunami resilience at the community level.

## 1. Introduction

**Figure 1.**
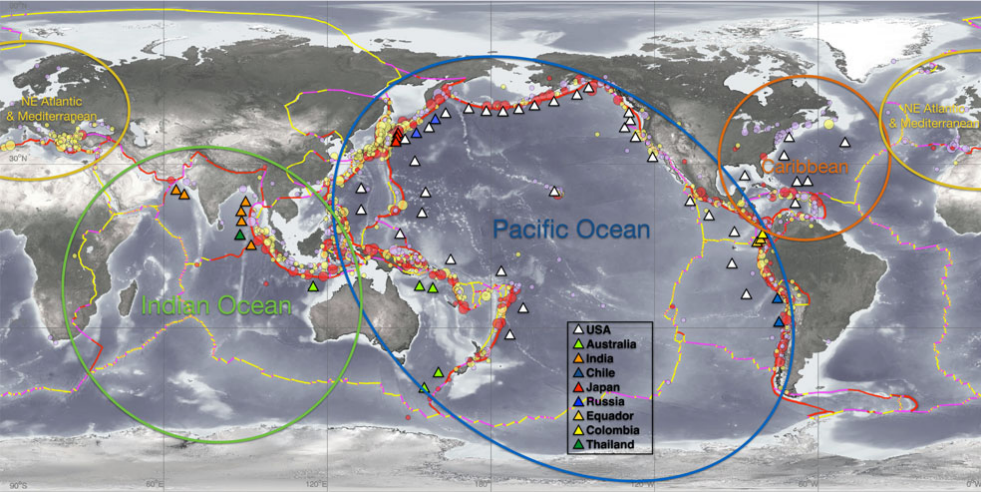
Global map of locations of historical tsunamis (circles) and DART stations (triangles) operated by nine nations. Tsunami generation locations since 1650 are indicated by circles where red indicates destructive tsunamis and yellow indicates tsunamis causing little damage. The larger the circle, the larger the earthquake. Coloured lines indicate major identified faults and plate boundaries. Subduction zones are identified as red lines. Ovals indicate four major regional tsunami warning systems that together comprise the global system.

A recent United Nations (UN) report estimates that every year about 60 000 people and $4 billion (US$) in assets are exposed to the global tsunami hazard [1]. Tsunamis inflict death and damage through violent, powerful flooding along the world's coastline (figure 1). Estimates of tsunami deaths and destruction will increase over time owing to population growth, migration to coastal areas, climate change and concentration of assets in ports [2,3]. The evolution of tsunami warning systems, however, has stronger correlation with destructive tsunamis than with assessing coastal risks. That is, after a damaging tsunami, the affected country takes action to protect its citizens and properties (table 1). In this review, we explore the evolution of science and technology used in tsunami warning systems, the evolution of their products using warning technologies, and offer suggestions for a new generation of warning products, aimed at the flooding hazard, to reduce future tsunami impacts on society.

**Table 1.** Evolution of tsunami warning systems AFTER major tsunamis.

tsunami	resulting tsunami warning system
1896 Japan	Japan-1941
1946 Alaska, USA	USA-1949
1952 Kamchatka, Russia	Russia-1954
1960 Chile	International Pacific Basin-1965
1964 Alaska, USA	French Polynesia-1965
2004 Sumatra, Indonesia	Global- 2007

Tsunamis are a series of long waves, generated by underwater earthquakes, landslides, slumps, volcanic eruptions, meteorological events and asteroid impacts, which violently flood adjacent and distant coastlines with devastating impact to coastal communities [4–7]. Tsunamis can be roughly classified as local, where coastal residents feel an earthquake and have only minutes before the tsunami begins flooding, or distant, where coastal residents do not feel the earthquake and have an hour or more before tsunami flooding commences. The evolution of tsunami warning systems began in the 1940s with a local tsunami warning system in Japan and a distant tsunami warning system in the USA. It then evolved in response to major tsunamis in 1946 Unimak, 1952 Kamchatka, 1957 Aleutian, 1960 Chile, 1964 Alaska, 1993 Japan, 1998 Papua New Guinea, 2004 Indian Ocean, 2010 Chile and 2011 Japan. The 2004 Indian Ocean tsunami, which killed over 235 000 people, was the watershed event that called for global action [8]. This evolution can be classified as (i) *Pacific; earthquake-centric* before the 26 December 2004 Indian Ocean tsunami and (ii) *global; tsunami-centric* after the world witnessed the horrific impacts of this deadly tsunami. Below, we chronicle the evolution of tsunami warning systems by country/region before and after the 2004 tsunami.

## 2. Pre-2004 Indian Ocean tsunami: Pacific; earthquake-centric

### (a) Japan (focus on local)

Because Japan has historically suffered the most tsunamis, it is only natural that Japan would be the first country to develop a tsunami warning system. Japan's development of tsunami science began in 1896 when a giant Sanriku tsunami, with run-up heights up to 38 m, claimed 22 000 lives. According to Shuto & Fujima [9], an article published in Japanese by an earthquake research group about the 1896 Sanriku tsunami identified an earthquake as the tsunami generator. Following this publication, there was an intense debate about the 1896 tsunami generation mechanism because of the extremely weak ground-shaking. Earthquakes of that type (weak shaking accompanied by strong tsunami) were later called tsunami earthquakes, and had become a major problem of all tsunami warning systems based on seismic measurements. Many researchers challenged the earthquake source hypothesis by proposing alternative mechanisms of an underwater volcanic eruption or landslide generators. Tsunami data recorded on a tide gauge indicating long wave periods resolved the debate, as only large fault motions could explain the formation of such a long period tsunami waves. Around 1910, Japanese researchers assumed that fault motions of earthquakes triggered tsunamis [9]. In 1933, the Sanriku area was impacted by another giant tsunami. Because the earthquake produced violent shaking, many coastal residents evacuated to high ground reducing the death toll to about 3000. With the success of the first early warning system, ‘if the ground shakes violently, evacuate,’ the Japanese earthquake research group proposed an earthquake-centric tsunami warning system. This was a practical approach, because seismic waves travel more than 10 times faster than tsunami waves, so the earthquake information could be used as a crude indicator of the approaching tsunami's strength. Japan's first instrumental warning system was established in September 1941 in Sendai, Japan, and was designed to quickly detect the earthquakes and warn people that a tsunami was imminent [9]. Using a seismometer at the Sendai Local Meteorological Observatory, an empirical forecasting chart was created, based on earthquake wave amplitude and the distance from the earthquake location to Sendai. If the combination of earthquake wave amplitude and distance were deemed dangerous, warnings were transmitted to the public through local radio stations and telephone calls to local police stations within 20 min of the earthquake origin time [9]. Many tsunami warnings were issued based on these crude estimates, empirically derived from four previous Japanese tsunamis, but the practice was to over-warn rather than to miss a tsunami. Five other regions in Japan adopted the Sendai model, but each region operated independently. In December 1946, the cabinet council of the Japanese government established a comprehensive plan for tsunami forecast and dissemination, which was based on the Sendai earthquake empirical seismic charts. In April 1952, the Japanese Meteorological Agency (JMA) began operating the system for all coastlines of Japan by establishing national standards.

Japan episodically refined its earthquake-centric approach to tsunami warnings. Following the 1983 Nihonkai–Chubu and the 1993 Hokkaido Nansei Oki tsunamis in the Sea of Japan, faster and more sophisticated seismic networks were established. By 1999, JMA could determine an earthquake's location, size and issue a tsunami warning within 3 min; along with tsunami wave height estimates based on pre-computed earthquake/tsunami scenarios [10]. The predicted tsunami wave heights were offshore estimates based only on the earthquake information. The problem with an earthquake-centric warning system is the inaccuracy of the tsunami forecast. As pointed out by Gusiakov [11], there is no direct relationship between earthquake magnitude and tsunami intensity. He concluded that there were other processes in tsunami generation that made earthquake magnitude a poor estimator of tsunami impact. The earthquake-centric warning method became vulnerable to inaccurate warnings (both over-warning and under-warning). The public was confused over the meaning of offshore wave heights, as a 3 m offshore tsunami height does not provide any information about the extent of flooding along the coastline. The consequence of both inaccuracy and misunderstanding by the public has led to inappropriate public response at a time of grave danger. It seems that the warning system had become an earthquake information provider, losing sight of the real tsunami hazard *violent flooding along the coastline*.

### (b) United States (focused first on distant, then local)

The first US tsunami warning centre was established in 1949 at the Honolulu Seismic Observatory following the death and destruction inflicted on Hawaii by the 1 April 1946 Alaska tsunami [12]. The USA adopted Japan's earthquake-centric approach and added a time of arrival capability for the world's first distant tsunami warning system. Tsunami travel times from the earthquake to Hawaii were based on the speed of the tsunami, which is proportional to the depth of water. Following the Pacific-wide 1960 Chile tsunami, the UN coordinated a Pacific-wide distant warning system for Pacific nations that began in 1965 and the USA offered to host the system, renaming the Honolulu Observatory the Pacific Tsunami Warning Center (PTWC). The US agency responsible for tsunami warnings was the National Oceanic and Atmospheric Administration (NOAA) and predecessor agencies, including the US Coast and Geodetic Survey and the Environmental Science Services Administration. The Pacific-wide tsunami warning system took advantage of the vast expanse of the Pacific Ocean and international cooperation among affected nations to create an ‘if you detect a tsunami, alert everyone’ approach [13]. Tsunami detectors were tide gauges around the Pacific ‘ring of fire’ and the telephone-based communications systems were tested monthly to ensure reliability in alerting all nations requesting tsunami services. Tsunami travel-time maps were provided to all participants, so times of tsunami arrival could be easily determined. This international cooperation enabled affected nations to have access to and the benefit of a large tsunami monitoring and arrival forecasting system for the cost of buying and operating a portion of the system. This shared cost system worked, but the inaccuracies of the forecasts led to a false alarm rate of 75% [12]. We define a false alarm here as a tsunami evacuation accompanied by a non-flooding tsunami. The inaccuracies and high false alarm rate were due, in part, to tsunami measurements at tide gauges being greatly influenced by local bathymetry. As such, using these data to predict the tsunami impact at other locations was of little quantitative value. In addition, the US earthquake-centric system suffered the same inaccuracy problems as Japan in using earthquake magnitude to estimate tsunami impact, i.e. inaccurate warnings.

The US established local tsunami warning systems following the 1964 Alaska tsunami, which was a local tsunami for Alaska and a distant tsunami for Hawaii and the US West Coast. This tsunami led to the 1967 formation of a local Alaska Tsunami Warning Center in Palmer, Alaska. In 1975, Hawaii experienced a local tsunami, killing two people, which led to the expansion of the Hawaiian seismic and tide gauge networks, enabling PTWC to operate as a local warning centre for Hawaii and a distant tsunami warning centre for the entire Pacific [12].

### (c) Russia (focus on local, contributed to Pacific-wide)

The 1952 Kamchatka tsunami led to the development of Russia's earthquake-centric tsunami warning system that included three local tsunami warning centres in the Kamchatka/Kuril Islands region. The centres were operated by the Hydrometeorological Service of the USSR and contributed to the UN Pacific-wide system for distant tsunami warnings while serving as local tsunami warning centres [14].

### (d) France (focus on distant, contributed to Pacific-wide)

The 1964 Alaskan tsunami led to the formation of the Polynesian Tsunami Warning Center in 1965 in Papeete, Tahiti. Because Tahiti is subjected to only distant tsunamis, it operated the same as the PTWC. The Polynesian Center also contributed to the UN's Pacific-wide system of seismic and tide gauge monitoring [14].

### (e) Tsunami warning products

Owing to the earthquake-centric nature of tsunami warning systems, emphasis was placed on earthquake monitoring and seismic data processing, not on coastal flooding. The warning centres were staffed with seismologists whose expertise was earthquake data analysis. Even though about 20% of tsunamis were generated by non-earthquake sources, the initial tsunami warning systems evolved into earthquake information centres, providing timely information on earthquakes. The fact that *tsunamis are a flooding hazard* was largely disregarded.

(a) Japan led the world in developing an ultra-sophisticated seismic network that, by 1995, could detect and size earthquakes in three minutes. Japan's system delivered two products—tsunami advisories and tsunami warnings. Tsunami warnings were divided into two categories: (i) *tsunami warning*, where waves up to 2 m were expected in some locations and (ii) *major tsunami warning*, where waves in excess of 3 m were predicted. Keep in mind that these were empirical predictions based on real-time earthquake magnitudes and historical tsunamis, not real-time tsunami observations. Also, the coastal community was left with the following dilemma: *how does a 2 m or 3 m offshore tsunami translate into flooding in my community?*
(b) United States and the Pacific-wide system also focused on earthquake monitoring. The three products offered by this earthquake-centric system were tsunami information (no tsunami expected), watches (there may be a tsunami, so stay tuned) and warnings (tsunami exists, evacuation recommended). These products, like the Japanese system, were based exclusively on earthquake information and subsequent tide gauge reports. By 1995, the level of warning was issued within 15 min of earthquake origin time for distant tsunamis and within 5 min of local tsunamis for Alaska and Hawaii. Products delivered were
  1. earthquake information—time, location and magnitude of the earthquake, which determined the tsunami warning level,
  2. time of arrival of tsunami, based on earthquake location and origin time and
  3. reports from tide gauges on the amplitude of the tsunami as it propagated.

Because the earthquake information was only a crude estimator of a tsunami's strength, there was little effort to predict the tsunami's impact on the coastline. Coastal communities were faced with the daunting task of deciding on tsunami evacuations in the face of great uncertainty. Erring on the side of safety, communities evacuated based primarily on the earthquake-centric warning level and historical tsunamis. Predictably, this practice led to many false alarms and unnecessary evacuations. For example, in 1986, a tsunami warning for Hawaii led to the evacuation of Waikiki, the dismissal of all state employees, and an ensuing traffic congestion that created a situation where *cars were gridlocked in evacuation zones*. The tsunami arrived on time, but only as non-flooding waves. The 1986 false alarm frustrated business owners, enraged the public and labelled the NOAA warning centre as inept. The State of Hawaii estimated this false alarm cost the state about $41 million (US$) [15] and led to the loss of credibility for tsunami warning products, which were disconnected from the flooding hazard. The 1986 false alarm was as influential as a major tsunami in the development of tsunami warning products useful to communities. In response to this costly false alarm, NOAA initiated a research programme in 1987 to develop a real-time *tsunami flooding forecast capability* based on deep-ocean tsunami observations (DART buoys). Within 25 years, a flooding forecast capability was created, tested and validated, and became part of NOAA's tsunami warning operations (§4 has more details of the full story).

## 3. Post-2004 Indian Ocean tsunami: global; tsunami-centric

The horrific 26 December 2004 Indian Ocean tsunami, which killed over 235 000 people, displaced 1.7 million across 16 countries and reached virtually all coastlines of the world [16], stimulated governments of the world into addressing tsunami hazards [17,18]. Many nations in the Indian Ocean did not even recognize the word ‘tsunami’ and none had tsunami preparedness programmes in place. Ignorance of the natural signs of a tsunami's presence led to inappropriate actions and decisions by nations, population centres and tourist destinations. The world's response to this terrible natural disaster was an unprecedented US$13.5 billion in international aid, including US$5.5 billion from the general public in developed nations. The 2004 tsunami, one of the top 10 deadliest natural disasters the world has recorded, will probably be best remembered for the expansion of the tsunami hazard reduction programme from just the Pacific Ocean to all coastlines of the world [19].

### (a) Japan (focus on local)

The Japanese earthquake-centric system was not changed following the 2004 tsunami. However, following the 2011 Japanese tsunami, JMA's evaluation of their performance during the 2011 tsunami recognized that the initial underestimate of the earthquake magnitude led to an initial underestimate of the tsunami [19]. As a result of these underestimates, the response to the warning, during a cold, snowy day, was also without urgency. When JMA received new information that the earthquake and tsunami were much larger, the affected populations did not receive many of their updates. Further, the JMA offshore tsunami amplitude information was confusing to the general public. That is: to what extent does a 3 m tsunami offshore flood my coastline? As a result of these findings, new procedures place emphasis on new observational capabilities, including offshore pressure sensors that report tsunami data in real time via an underwater cable. Japan plans to use offshore tsunami measurements to more accurately forecast tsunami coastal impacts [20]. In addition, Japan deployed three DART buoys off its coast, and shares their tsunami data with all nations of the global system [21].

### (b) United States (focused first on distant, then local)

The over-warning problem in Hawaii led the USA to develop a more accurate method of forecasting tsunami flooding through direct measurements of the tsunami in the deep ocean, free of coastal influences. Data from these tsunami detectors were assimilated into forecast models to predict the coastal flooding of an approaching distant tsunami hours before arrival. These real-time, deep-ocean tsunami detectors, termed DART buoys, improved the accuracy of tsunami forecasts. NOAA scientists made the first experimental forecast for the November 2003 tsunami generated in the Aleutian Islands using data from two DART buoys assimilated into forecast models. The results indicated that the tsunami would be only 0.3 m in Hawaii, and were used, in part, to avoid an unnecessary evacuation or false alarm [22]. This experimental forecast was reported at the 2005 UN meeting where a global tsunami warning system was discussed. The USA offered to share this tsunami flooding forecast technology with Indian Ocean nations. The Indian Ocean nations quickly accepted the offer and designed the Indian Ocean tsunami warning system after the flooding focused, tsunami-centric US system. By 2015, the Indian Ocean tsunami warning system was operational with eight DART buoys owned and operated by Australia, India and Thailand. In addition, regional warning systems in the Caribbean adopted the US approach (figure 1) with the US operating seven DART buoys in the region. Using tsunami data from DART buoys instantly standardized the global tsunami observational network, which paved the way for standardized procedures and products [23].

**Figure 2.**
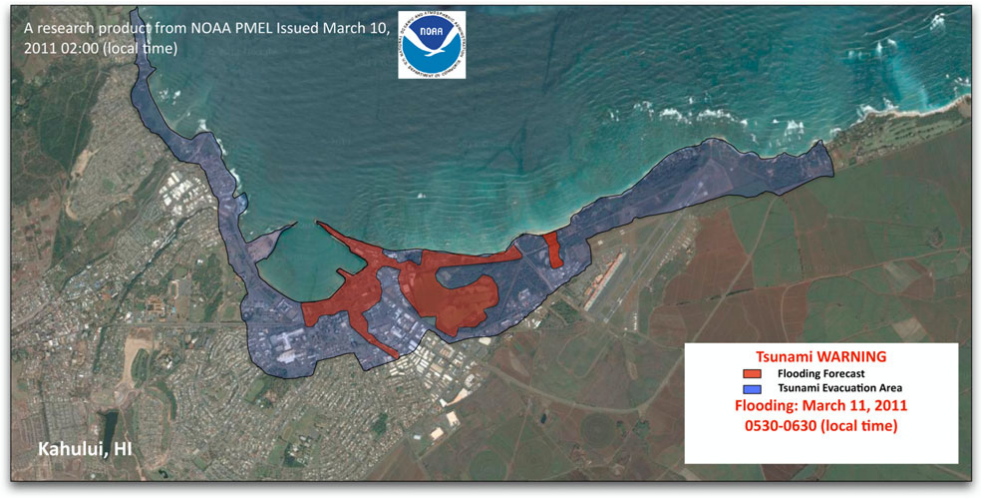
Tsunami flooding map for Kahului, Hawaii, from the 11 March 2011 Japanese tsunami. Blue areas indicate tsunami evacuation zones while red areas indicate flooding forecast for this tsunami.

Meanwhile, experimental tsunami forecasts for the Kuril Islands (November 2006 and January 2007), Tonga (May 2006), Solomon Islands (April 2007), Peru (August 2007), Chile (November 2007, 2010) and American Samoa (September 2009) were made for 12 US coastal communities [24–26]. When scientists compared the experimental forecasts with sea-level data from coastal stations for the eight tsunamis, they found that the forecasts were within 80% agreement with tide gauge records [27]. For the 2011 Japanese tsunami, data from four DART buoys near Japan were used to produce the world's first forecast of tsunami flooding for Kahului (figure 2) and other locations in Hawaii, *6 h before the tsunami struck* [28]. An evacuation order was issued, and lives were saved. In 2013, this tsunami flooding forecast system became operational at both NOAA tsunami warning centres.

### (c) Russia (focus on local, contributed to Pacific-wide)

Russia continued to participate in the Pacific-wide system coordinated by the UN. Russia also deployed DART buoys in 2010 and 2012, and shares their data with other nations. Data from one Russian and three US DART buoys provided an accurate forecast of the 2011 Japanese tsunami for US coastlines. This example of unselfish sharing of vital data between Russia and the USA is a model for international cooperation.

### (d) France (focus on distant, then local contributed to Pacific-wide)

The Polynesian Tsunami Warning Centre continued to contribute to the UN Pacific-wide system of seismic and tide gauge monitoring. In addition, the National Tsunami Warning Centre (CENALT, France, hosted by the Atomic Energy and Alternative Energies Commission) was established in 2012 as part of the North Atlantic and Mediterranean tsunami warning system.

### (e) Global system

The UN-coordinated effort, in place in the Pacific before the 2004 Indian Ocean tsunami, was expanded to the other tsunami-threatened coastlines. The global system, comprised regional warning centres in the Indian, Atlantic and Pacific oceans, and the Caribbean seas [23], has about 60 standard deep-ocean tsunami detectors that provide data, freely shared among nations, for forecasting tsunami impacts (figure 1). One characteristic of the tsunami warning systems in the Indian Ocean and Mediterranean Sea is the introduction of multiple regional tsunami service centres providing warning products to all nations of the affected area. For example, there are three regional centres in the Indian Ocean: (i) Australia, (ii) India and (iii) Indonesia. Australia and India operate DART stations (figure 1) and use the US tsunami-centric approach for warning products. Indonesia, in cooperation with Germany, uses an earthquake-centric approach, similar to Japan, for local tsunamis in Indonesia. The Indonesian/German system uses earthquake information to activate pre-computed simulations to issue warnings. The warnings are then updated once a coastal tide gauge has detected the tsunami. All three regional service providers supply warnings to all Indian Ocean nations. Thus, all Indian Ocean countries benefit from the operations of three providers. The Mediterranean Sea tsunami warning system uses an earthquake-centric approach, as there are no DART stations currently in the Mediterranean Sea. Local and distant warnings are based on earthquake information only. To verify the existence of a tsunami, the Mediterranean system uses coastal tide gauges. Like the Indian Ocean tsunami warning system, five nations (France, Greece, Italy, Portugal and Turkey) will provide regional tsunami warning services to subscribing nations surrounding the Mediterranean Sea. The UN's Intergovernmental Oceanographic Commission (IOC) has a plan that calls for international standards for all regional warning systems’ procedures and products to ensure interoperability and understanding by global citizens [23]. When implemented, the global tsunami warning system will serve as an example of international cooperation for a flooding hazard that does not recognize national boundaries.

### (f) Tsunami warning products

**Figure 3.**
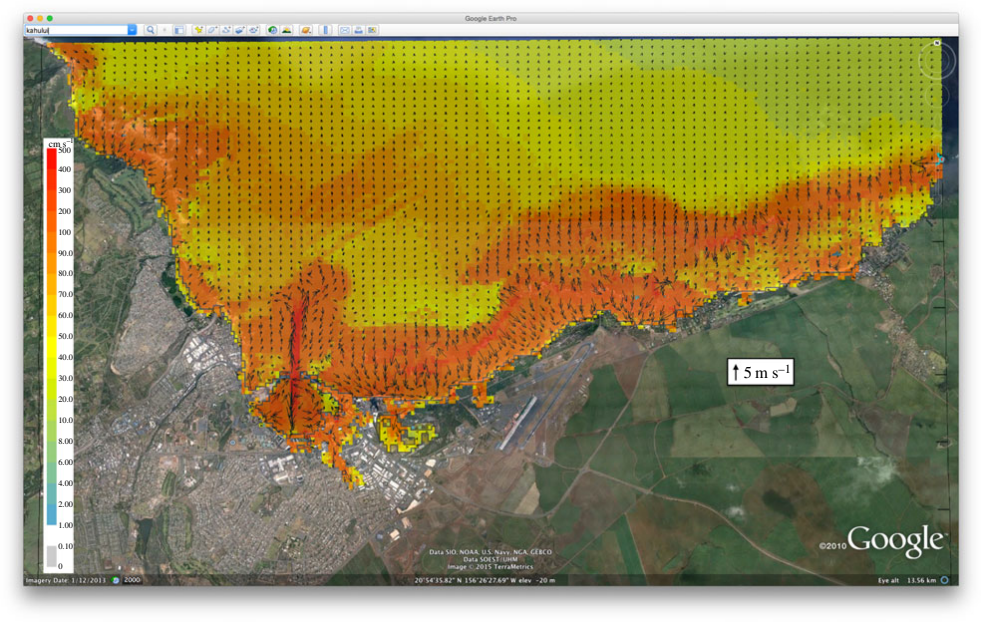
Maximum tsunami-induced currents in harbour map for Kahului, Hawaii from the 11 March 2011 Japanese tsunami. Arrows indicate direction of flow, while arrow length represents current speed.

With the development of the global, tsunami-centric warning systems, new products associated with the tsunami-flooding hazard are now possible [29]. One possibility is products for ports and harbours. Operators of ports and harbours are required to work in a potentially hazardous area, yet most large ports lack tsunami evacuation plans. This problem is due, in part, to the multi-jurisdictional aspects of port operations. Further, no tsunami warning products are available to guide port operators under tsunami siege. Tsunami-induced currents cause damage to ports in at least three ways. First, current velocities exceed design limits for pier pilings, causing collapse of port piers and associated infrastructure such as cranes, utilities and containers (some ports can have up to 10 000 containers at a time). Second, current velocities exceed mooring line strength, allowing ships to become free floating battering rams which can destroy port infrastructure, block exits for other ships and become sources of fires. Third, combined port destruction, flooding and fires can kill and injure port workers and ship personnel. By far, the most destructive element is ships adrift in a restricted port. Recent studies have shown that current velocities vary greatly within a port, revealing that low current areas could be safe havens for ships [30]. If there is inadequate time to evacuate ships, port operators could move ships to these safe havens to minimize port damage. To do this, port operators need to know where the high- and low-velocity areas are located (similar to inundation maps) and real-time current forecasts to guide decision-making. Offshore oil terminals also need tsunami-induced current velocity forecasts to avoid oil spills, a major potential hazard for the environment and as a source of fires. Existing tsunami flooding forecast models could produce such current products [31], as illustrated in figure 3. In figure 3, arrows represent the maximum current velocities and direction predicted for the 2011 Tohoku tsunami. The length of the arrows represent current speed, i.e. the longer the arrow, the greater the current speed. Within the port of Kahului, there is a forecast of 10-knot (5 m s^−1^) currents, and offshore currents are forecasted to be in excess of five knots. This type of information would be used by port operators to evacuate the port and recommend areas to avoid offshore until the tsunami has subsided. For this model technology, model currents speeds matched observed current speeds with 70% accuracy [31,32]. Tsunami-induced current warning products for ports and harbours will lead to appropriate evacuations of people and ships from hazardous areas and will minimize the disruption to port operations. For example, Borrero *et al.* [33] found that if the California ports of Long Beach and Los Angeles were closed for 1 year owing to tsunami damage, the impact on the US economy would be US$ 43.5 billion [33]. It is in everyone's interest to have port operation resume as quickly as possible following a tsunami.

**Figure 4.**
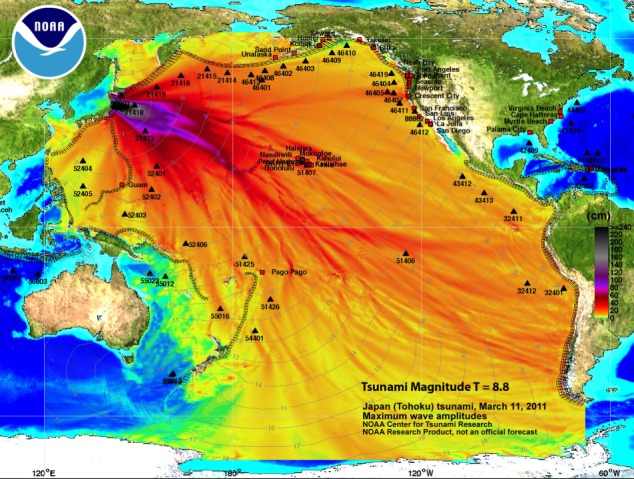
Tsunami magnitude (total tsunami energy) product added to the tsunami maximum amplitude map from the 11 March 2011 Japanese tsunami.This warning product will give the public detailed information on which coastline may be affected by the tsunami and a total energy estimate.

Another tsunami warning product that is possible with the establishment of a deep-ocean tsunami detection array is a tsunami magnitude scale based on accurate real-time tsunami energy estimates. The total energy transmitted by tsunami waves is one of the most fundamental quantities for quick estimation of the potential impact of a tsunami. Recognizing the importance of such scale, Kajiura [34] suggested several means of estimating the tsunami energy in 1981. However, he concluded that the direct tsunami measurements (only tide gauges at the time) were not adequate to estimate tsunami energy in a post-event mode, let alone in a real-time mode. The seismic source parameters’ uncertainties and the fact that only a small fraction (around 0.1%) of the energy released from an earthquake is transferred into the ocean to generate a tsunami [28] make the seismic magnitude too crude an estimate for the tsunami energy. Kajiura [34] estimated a factor of 2–3 to be the best accuracy possible with tide gauge and seismic data, which made the energy scale impractical at the time. While seismic analysis of an earthquake has improved since 1981, the factor of 2 accuracy for the earthquake parameters is still the limiting factor for the tsunami energy estimates, especially in the real-time tsunami warning situation. DART data provide new opportunities for direct estimate of the tsunami energy quickly and accurately. The DART-inverted source quantifies the amount of energy that propagates outside the source area in the form of long gravity waves that define coastal impacts. As such, it gives all affected coastlines an estimate of the tsunami's potential impact and an accurate threshold for action. Tang *et al.* [28] showed that the tsunami energy based on one DART measurement was available within 56 min from the time of generation during the 2011 Japanese tsunami. This value was within 20% of the final estimate of the tsunami energy based on thorough analysis of all DARTs for this event, demonstrating that the tsunami energy can be estimated accurately and quickly. Using this methodology, an example of an energy product is shown in figure 4. In figure 4, the maximum tsunami amplitude forecast map from the 2011 Japanese tsunami shows the distribution of tsunami amplitudes (a proxy for tsunami energy) along with the tsunami magnitude, a representation of the total tsunami energy. At a glance, the public can see the overall intensity of the tsunami (very large) and the distribution of the energy along all coastlines of the Pacific Ocean. Note that most coastlines will not be affected. The coastal areas that will be affected can have coastal flooding maps (figure 2) and tsunami-induced current maps (figure 3) to guide local evacuation and port operation decisions. With more DART buoys, the time to calculate the tsunami energy estimate will be reduced. A tsunami energy scale should be developed to communicate tsunami energy levels (and thus, concerns) to the public. Tsunami flooding products will assist coastal communities in dealing with the tsunami, aid search and rescue operations, and guide in the community's long-term recovery.

## 4. Evolution of tsunami warning technology

As described in §§2 and 3, with the establishment of a deep-ocean tsunami detection system, tsunami warnings are evolving from crude earthquake-centric products to accurate tsunami-centric flooding products. Currently, there are three technologies that can provide offshore tsunami observations in real time for use in warning operations: (i) DART, (ii) cabled observatories and (iii) differential GPS buoys.

(1) DART: this buoy-based technology offers the following advantages: can serve as a local and distant tsunami detection system, is portable, so array locations can be easily changed, standardized sensors, shared data with other countries in real time, represents a distributed system so one failure will not affect the other DARTs and initial low-cost investment. Disadvantages include high emergency maintenance costs owing to ship-related costs and buoy locations must avoid high ocean currents and seamounts. The DART easy-to-deploy model can help reduce maintenance costs, because a smaller vessel (i.e. fishing boat) can be used in emergency repair/replacement. Approximate cost is about $0.5M/DART station.
(2) Cabled observatories: this cable-based system has the following advantages: can serve as a local and distant tsunami detection system, uses the same sensor as used in DARTs, so measurements are compatible, four developed countries have installed cabled observatories (Canada, Japan, Oman and the USA) and can support a dense network of pressure sensors. The disadvantages include: single point of failure should the cable fail and high initial costs. For example, Japan is installing a 1000 km cabled observatory with 164 pressure sensors at an estimated initial cost of US$ 500M.
(3) Differential GPS buoys: this buoy-based technology offers the following advantages: can serve as a local tsunami detection system, is portable so array locations can be easily changed, represents a distributed system, so one failure will not affect the other GPS buoys and initial medium-cost investment. Disadvantages include: must be located close to the coast (within line of site) to communicate with GPS base stations, non-standard sensor, so measurements are not compatible with DART and cabled observatories sensors, and high emergency maintenance costs owing to ship-related costs. Approximate cost is about $3M/GPS station.

In summary, nine nations (Australia, Chile, Colombia, Ecuador, India, Japan, Russia, Thailand and the USA) are operating 60 DARTs (figure 1) for their warning requirements and sharing data globally in real time, four nations (Canada, Japan, Oman and the USA) are investing in cabled observatories for tsunami research and are not sharing data globally, and Japan is investing in GPS technology for operations and is not sharing data globally.

Now that the technology exists to make real-time offshore tsunami measurements, how will these data be used to mitigate the impact of future tsunamis? The USA has established an operational tsunami flooding forecast capability that could become a basis of an international standard [19]. This tsunami flooding capability can be used by all tsunami-threatened nations through new web-based software technology, including cloud computing. The concept would be to integrate the existing US system through a web-based software package. Analogous to commonly used software packages, the tsunami forecast system could be used by all nations with modest training. Trained technicians could use the forecast system to examine past tsunamis, participate in real-time tsunami warnings and simulate hypothetical scenarios.

The elements are in place to create such a software package, building upon tsunami flooding training activities such as Community Model Interface for Tsunamis (ComMIT) [35]. Following the Indian Ocean tsunami of 26 December 2004, the UN established a coordinating group for the Indian Ocean Tsunami Warning and Mitigation System. This group recommended the establishment of a web-based community tsunami-flooding model. It was envisioned that this would be the primary avenue to transfer modelling expertise and capability to, between and within Indian Ocean countries. It would provide a platform for the construction of tsunami inundation maps for different earthquakes, as well as real-time tsunami forecast applications and, thus, would be a critical tool for building tsunami-resilient communities in the region.

ComMIT interface provides access to US flooding forecast tools and capabilities via web-enabled interface that links users into distributed tsunami forecast community. In this context, ComMIT refers to a community of users, rather than developers of commonly used open-source model of collaboration. ComMIT focuses on developing and using a local tsunami flooding model. Complexities and uncertainties of tsunami source definitions and ocean-wide tsunami propagation forecasts are separated from the local flooding hazard estimates and can be handled by regional operational tsunami services. The interface has access to these operational resources via Internet connection. At the same time, ComMIT users have full control of local data and local forecast results. This distributed approach has built-in maintenance of standards for developing consistent and compatible flooding forecast capabilities. ComMIT modellers use the same tool with the same input data, so results can be benchmarked, verified and inter-compared easily.

Since 2007, about 17 week-long training sessions have been held in Pacific and Indian ocean nations with over 300 students being trained, with support from UNESCO, USAID and AusAID. Tsunami flooding models that have been developed using the ComMIT tool now cover most of the coastlines of the Indian Ocean and a good portion of the southwest Pacific islands.

**Figure 5.**
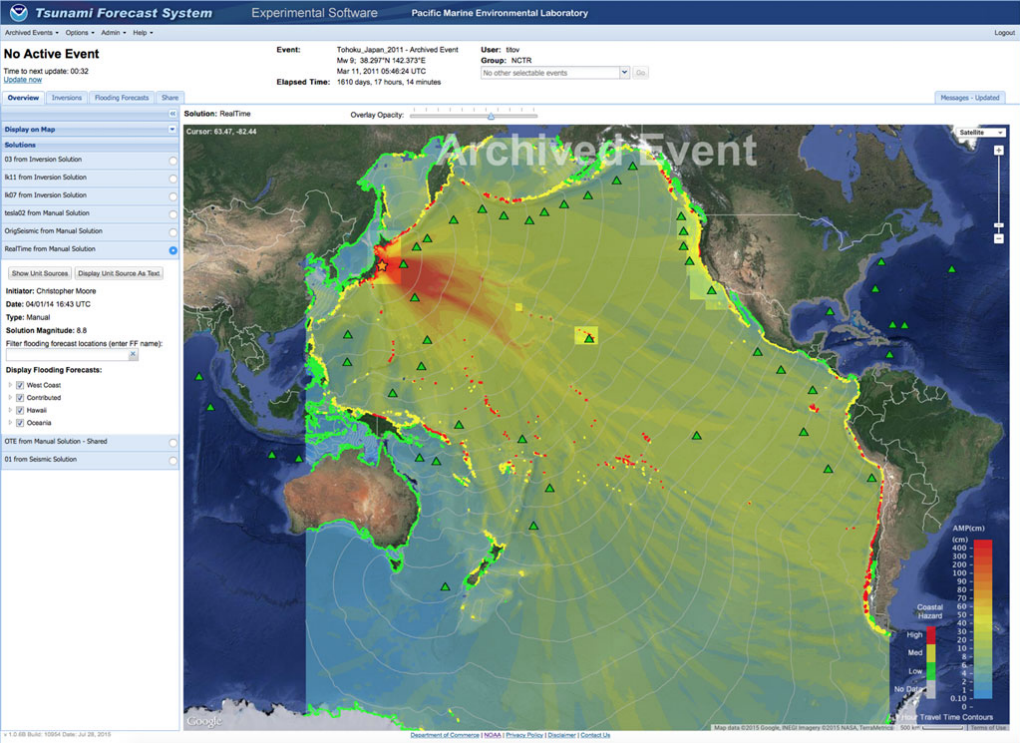
Screenshot of Tsunamiweb—a tsunami forecast software package.

A prototype web-based version that works in tandem with the ComMIT software package, named Tsunamiweb, aka Tweb (figure 5), is presently being tested at NOAA. Tsunamiweb can easily share ComMIT-produced tsunami flooding maps (figure 2) and tsunami-induced currents in harbour maps (figure 3) and energy maps (figure 4). With modest training, any nation can establish a tsunami flooding capability through Tsunamiweb. Such a software package could be supported using several options, including

1. as part of its international tsunami warning products, NOAA could provide vital information about an impending tsunami to be used as input to the international tsunami forecast package (Tsunamiweb);
2. as an international effort, nations could share the costs by providing vital DART data and/or other support through the UN's IOC or the World Meteorological Organization; or
3. paying service fees to a commercial enterprise.

An international panel of experts should establish and maintain strict scientific and software standards to ensure the integrity and credibility of the web-based systems. Initial tsunami modelling standards have been already established in reference [36]. Such an approach would help the UN achieve its goal of interoperability and standard tsunami warning products throughout the world. As such, international cooperation could reduce the impact to all nations while building capacity at low initial and continuing costs. Embracing this web-based software technology now will assure nations of better resilience from the next tsunami.

## 5. The future

In our opinion, coastal communities would be well served by receiving three *standardized, accurate, real-time* tsunami warning products, namely (i) tsunami energy estimate (figure 4), (ii) flooding maps (figure 2), and (iii) tsunami-induced currents in harbour maps (figure 3), to minimize the impact of tsunamis. Such information would arm communities with vital guidance for evacuations and port operations, rescue operations and long-term recovery. Delivery of such information in real time would be possible through a web-based forecasting system as described in §4. The advantage of global standardized products delivered in a common format is efficiency and accuracy, which leads to effectiveness in promoting tsunami resilience at the community level. Another advantage is the cost savings that allow assets to focus on community needs, such as land use management, education, training and drills. It also allows researchers to focus on the flooding needs of the tsunami-threatened community, such as building codes and barriers to minimize the impact of future tsunami flooding [37]. Finally, we emphasize that education of coastal residents and visitors must be part of any tsunami resilience plan. For local tsunamis, education is by far the most cost-effective investment [38,39]. If you are on the coast and feel the earth shake for more than 1 min, see rapid changes in sea level or hear a roaring sound from the ocean, a natural warning has been issued.
